# Small-scale urban design interventions: A framework for deploying cities as resource for mental health and mental health literacy

**DOI:** 10.3389/fpsyg.2023.1112209

**Published:** 2023-03-20

**Authors:** Helena Müller, Jonas Rehn-Groenendijk, Anna Wasmer

**Affiliations:** ^1^Department of Social and Cultural Sciences and Social Work, Darmstadt University of Applied Sciences, Darmstadt, Germany; ^2^Department of Design, Darmstadt University of Applied Sciences, Darmstadt, Germany; ^3^Department of Civil Engineering, Darmstadt University of Applied Sciences, Darmstadt, Germany

**Keywords:** urban environment, restorative cities, urban mental health, mental health literacy, salutogenesis, psychosocially-supportive design, evidence-based design, participation

## Abstract

With roughly half of the global population living in cities, urban environments become central to public health often perceived as health risk factors. Indeed, mental disorders show higher incidences in urban contexts compared to rural areas. However, shared urban environments also provide a rich potential to act as a resource for mental health and as a platform to increase mental health literacy. Based on the concepts of salutogenesis and restorative environments, we propose a framework for urban design interventions. It outlines (a) an output level, i.e., preventive and discursive potentials of such interventions to act as biopsychosocial resources, and (b) a process level, i.e., mechanisms of inter- and transdisciplinary collaboration of researchers and citizens in the design process. This approach aims at combining evidence-based, salutogenic, psychosocially-supportive design with a focus on mental health. Implementing low-threshold, resource-efficient options in the existing urban context brings this topic to the public space. Implications for the implementation of such interventions for citizens, researchers, and municipality stakeholders are discussed. This illustrates new directions of research for urban person-environment interactions, public health, and beyond.

## 1. Introduction

About 55% of the global population is living in cities and this trend is expected to rise up to 68% by 2050 (United Nations, 2018). This renders urban environments central to public health. Often, urban environments are perceived as health risk factors due to, e.g., noise, pollution, crowding, and anonymity (Gruebner et al., 2017). Indeed, mental disorders show higher incidence in urban contexts compared to rural areas, generally increasing, e.g., due to the COVID-19-pandemic (Fofana et al., 2020) as well as due to climate change (Hickman et al., 2021). Mental health problems such as anxiety disorders, major depression, and schizophrenia occur up to 56% more frequently in urban areas than in rural areas (Peen et al., 2010; Prina et al., 2011). With regard to higher incidences of schizophrenia in urban contexts, research suggests that these are associated with increased social stress (Lederbogen et al., 2011). In addition, phenomena such as homelessness or crime are more prevalent in urban areas compared to rural areas (Adli, 2017). Worldwide, mental disorders affect one in eight adults (WHO, 2022). Despite the diversity and increasing prevalence of mental disorders, the topic of mental health continues to be plagued by misconceptions and stigmas (Angermeyer et al., 2013). Such stigmatization often leads to an aggravation of the symptoms and increases the psychological strain for those affected and their relatives (Sickel et al., 2014). Studies show that mental health literacy, i.e., knowledge that helps to recognize, manage, or prevent mental disorders (Jorm et al., 1997), can promote attitudes toward people affected by mental disorders, especially for depression (Svensson and Hansson, 2016). However, levels of mental health literacy are only low to moderate worldwide (e.g., Jorm, 2012; Dang et al., 2020). At the same time, provision of psychotherapy services is precarious in the US (Andrilla et al., 2018) as well as in Europe (Priebe and Wright, 2006; Bundespsychotherapeutenkammer, 2018) with long waits for a first consultation and strong variation between urban and rural areas. This situation calls for low-threshold possibilities not only in treatment, but more importantly in the prevention of mental disorders – including mental health literacy. As mental health is still a topic mainly discussed behind closed doors (i.e., in doctors’ offices, clinics, consulting rooms, and lecture halls), we see shared urban environments as opportunities to decrease stigmatization of mental disorders through communication and psychoeducation. This is in line with other recent endeavors such as the development of mental health first aid programmes (e.g., Kitchener and Jorm, 2008), which aim at teaching people of the general public how to identify and understand signs of mental health problems in order to provide initial help.

The relationship between the built environment and health and wellbeing has been subject to research in a variety of disciplines for decades (e.g., Rubin et al., 1998). This research has been largely prompted by Ulrich’s (1984) influential article on the window view from a hospital room and its beneficial effect on the recovery from surgery when facing a tree instead of a brick wall. While Ulrich explicitly pointed to the less monotonous character of the nature scene as a confounding factor, his article can be seen as a corner stone for the biophilic design paradigm (e.g., Kellert et al., 2008). Further concepts on the relationship between society and space originate mainly from social geography (constructivist and structuration theory perspectives; Backhaus and Müller-Böker, 2006) and include questions about preconditions for successful appropriation of space (Werlen, 2000), ways of appropriating space, and rules of appropriation. From a psychological point of view, space appropriation represents the (experienced) change of a space that occurs through mental (e.g., remembering) or physical (e.g., painting) activities (Rump and Richter, 2009). Thus, appropriation transforms an initially neutral, unfamiliar environment into one that is personally meaningful (Steg and de Groot, 2018). Influencing our built environment meets basic human needs for autonomy and competence (e.g., Vollmer et al., 2020). In terms of public space, appropriation behavior increases attachment to these places (Rioux et al., 2017), which in turn is associated with increased social participation in the neighborhood (Mihaylov and Perkins, 2013).

Against this backdrop, there are no prototypical cities that can be designed perfectly for everyone. Rather, it is a matter of taking into account the specific needs of local residents and visitors and creating opportunities for participation and appropriation (Vollmer et al., 2020). Therefore, we argue that urban environments not only pose risks but also act as an insufficiently considered resource for mental health of citizens and to address mental health issues in the public space in a low-threshold way (van der Wal et al., 2021). Whereas many other frameworks (e.g., Vollmer et al., 2020; Roe and McCay, 2021; Bornioli and Subiza-Pérez, 2022) eventually advocate for long term urban planning approaches to foster mental health, we aim at shedding light on smaller and easily implementable design interventions that make use of the given surroundings and its inherent potentials. Without neglecting the profound utility of approaches that are more comprehensive and include long term urban planning processes, this complementary concept allows cities to quickly address mental health and mental health literacy in a cost and time effective manner. In this article, we outline a framework for putting this idea into practice.

## 2. Theoretical background

Urban mental health is a complex topic that asks for interdisciplinary collaboration. In this article, we aim to integrate perspectives from (a) environmental and health psychology, (b) design research, and (c) urban planning. To that aim, we first draw on these disciplines’ theoretical stock, before we develop a conceptual framework for deploying cities as resource for mental health and mental health literacy in terms of output and processes. Finally, we discuss the benefit of the framework in theoretical and practical implementation.

### 2.1. Psychological perspectives on urban mental health

The relations between urban environments and mental health are complex, including both resources (e.g., access to healthcare and education) as well as stressors (e.g., noise and crowding) (cf. Vollmer et al., 2020). Following a fundamental perspective in environmental psychology, the built environment affects human health on different levels (Kirsty et al., 2018; Wasfi and Kestens, 2021). These impacts range from physical aspects (e.g., traffic safety, reaction to heat; Tong et al., 2021) to psychological processes (e.g., stress due to crowding, loneliness due to anonymity; Knöll et al., 2018) to behavioral aspects (e.g., car usage due to low walkability; Sugiyama et al., 2019). At the same time, humans are not fully at the mercy of the environments but have opportunities – at least to a certain extent – to shape the environments we engage with, for example through relocation or space appropriation (Steg and de Groot, 2018). Concerning mental health, this leads to two different effects (Gruebner et al., 2017): causal effects (i.e., direct influences of social-spatial environment of the city on people) as well as selective processes (i.e., features of the city, such as supply structures and job opportunities, that favor the influx of certain groups of people). Both effects shape the interaction between people and their (urban) environments.

More specifically, in environmental psychology, a prominent line of research on environments and health concerns *restorative environments* (Hartig, 2004). These comprise the idea that natural as well as built environments can help restore depleted resources consumed during the day and reduce stress, which can in turn support physical as well as mental health (Roe, 2010; Weber and Trojan, 2018; Scopelliti et al., 2019). This represents an additional function of urban space apart from providing supply structures, housing and working. To date, there is a strong focus on the positive significance of the natural environment in cities (e.g., green spaces, Astell-Burt et al., 2021; urban trees, Marselle et al., 2020; water, White et al., 2021; for an overview, see Hartig, 2021), with weaker emphasis on the built environment (Bornioli and Subiza-Pérez, 2022). From there, we can extract two characteristics that amplify the importance of public space as restorative environments: (a) reduced action range (e.g., due to pandemic lockdown, health or financial constraints; Nieuwenhuijsen, 2020), (b) lacking resources for private recreational opportunities and spaces (e.g., garden, balcony; Astell-Burt et al., 2014). While reduced action range requires a decentralization of recreational potential across the city, low-income and high-density areas underline the importance of accessibility of recreational public spaces. In line with this, design measures in the urban context can reduce or exacerbate social inequalities, as impressively demonstrated in an early study by Moore (1985).

Drawing on concepts from health psychology such as the *Salutogenic Model of Health* (Antonovsky, 1979) can help to further develop the idea of urban contexts as restorative and health-promoting environments. In accordance with this model, the aim is not to avoid stress and other burdens and risk factors in general, but to find ways for people to remain healthy despite high stress levels (von Lindern et al., 2017). Following Antonovsky’s concept, health promotion is to be understood as a continuous process whose endeavor is to move closer toward health on a continuum between illness and health (cf. Bengel et al., 1998). Of decisive importance here is the sense of coherence postulated by Antonovsky, which can be divided into the three aspects of “comprehensibility,” “manageability,” and “meaningfulness” (Bengel et al., 1998, p. 28 f.). As also taken up later under the term *Salutogenic Design* (Dilani, 2005), the built environment can have a significant influence on the expression of this sense of coherence (e.g., Rehn, 2019).

The distinction of different types of health, e.g., mental and physical, is rooted in the *Biopsychosocial Model of Health* (Engel, 1977), which is an alternative to the biomedical understanding of health. Coming from systems theory, the salutogenic and the biopsychosocial approach can be merged to the intention of developing health-promoting measures (instead of disease-fighting) taking into account biological, psychological, and social elements (instead of biological only). Although this paper focuses primarily on mental health, this cannot be clearly distinguished from other facets of a holistic concept of health (cf. WHO, 1946). Findings from fields such as psychoneuroimmunology (e.g., Schubert, 2011), embodiment (Barsalou et al., 2003) and psychosomatics (e.g., Davidson et al., 2003) suggest a systemic interdependence in which mental wellbeing is both a manifestation and a cause of health.

### 2.2. Design principles for promoting health and wellbeing

The effect of the urban context on health and wellbeing can be categorized into at least four different influencing factors (Baumgart et al., 2018): (1) the built environment, (2) social factors (e.g., social integration and mobility), (3) political administrative factors (e.g., density of close healthcare provision), and (4) symbolic factors (e.g., cityscape). While all of these categories pose important potentials and leverages for addressing health through urban interventions, this paper and the presented framework focus primarily on the built environment. Still, as all four categories are interrelated, the built environment can influence, e.g., symbolic factors of a city (its “look and feel”) or social factors by providing affordances to facilitate social integration and reduce disparities (e.g., Bagnall et al., 2017). Apart from that, a growing body of literature points to direct effects of the built environment on health and wellbeing, e.g., through increasing physical activity (e.g., Center for Active Design, 2010), reducing stress (Ulrich et al., 2008; Knöll et al., 2018), or increasing accessibility and inclusion (Amin, 2018).

As a basis for architecture, design, and urban planning, scientific evidence has gained relevance in recent years related to an evidence-based design approach (Hamilton, 2003; Malkin, 2008; Devlin, 2014). This is where the later presented framework picks up by applying a broad spectrum of scientific evidence from psychology, urban planning, and design research in an interdisciplinary collaboration. The results obtained in such research-driven design approach can inform a human-centered design process (Visocky O’Grady and Visocky O’Grady, 2017).

While already early work from the 19th century draws interrelations between design and health (e.g., Nightingale, 1860, 1863), Ulrich’s (1984) seminal paper on views from a hospital window can be seen as the starting point of extensive exploration of the impact of design on health and wellbeing. Continuously, frameworks elaborated on this idea emphasizing partly different design aspects such as therapeutic landscapes (Gesler, 1993), psychosocially-supportive design (Ulrich, 1997), salutogenic design (Dilani, 2005), healing environments (Dijkstra, 2009), and biophilic design (Kellert et al., 2008; Ryan and Browning, 2020). Whereas these frameworks mainly focus on direct effects on health and wellbeing, other approaches extend this scope by addressing how design can influence health behavior (e.g., Fogg, 2003; Lockton et al., 2010; Michie et al., 2014; Rehn, 2018). In some cases, this includes aspects of gamification and approaches of augmented reality (e.g., Knöll et al., 2014; Halblaub et al., 2015). Some of these paradigms represent the theoretical basis upon which a number of design tools and guidelines for urban and public design were created. These include the Active Design Guidelines (Center for Active Design, 2010), the Building Healthy Places Toolkit (Urban Land Institute, 2015), the Assembly Civic Design Guidelines (Center for Active Design, 2018), and the Great Place Guide (Australian Capital Territory, 2020).

One of the few models that focus specifically on mental health promotion in cities is the *Restorative Cities Framework* by Roe and McCay (2021). The model links numerous established theories and paradigms from design and architecture research (e.g., biophilic design and salutogenic design) and psychology (e.g., *Attention Restoration Theory*; Kaplan and Kaplan, 1989). It comprises seven characteristics that distinguish a city as “restorative”: inclusive (i.e., equal access to health-promoting services, including, e.g., those with lower income), green (i.e., integrating nature into the urban core), blue (i.e., access to water), sensory (i.e., engage all five senses), neighborly (i.e., support social cohesion), active (i.e., promote cognitive and emotional wellbeing through movement), and playable (i.e., provide opportunities for creativity and play for all ages). This reflects the components of a biopsychosocial understanding of health (Engel, 1977), whose holistic approach forms the backdrop of this article.

Another design paradigm that poses a pillar upon which the later illustrated framework is built relates to *experience design* (XD) – an approach that is usually not applied to city planning and urban design but rather to retail and web design. One of the fundamental goals of experience design is to create coherent end-to-end experiences that aim at achieving a specific goal or satisfy one or more needs (e.g., Risdon et al., 2018). While the field of experience design uses terminology such as “channels,” “touchpoints,” and “service blueprints” whose explanation would go beyond the scope of this paper, the notion of “journey” poses a useful perspective for urban design in the context of mental health promotion. As Risdon et al. (2018 p. 88) point out, journeys can be operationalized as “the conceptual trip a person embarks upon to achieve a goal or satisfy one or more needs.” With respect to urban design interventions, the conceptual link between physically separated concepts through a journey that can be adapted by users based on their time and willingness to continue can increase the effectiveness of the sum of all parts. In practice, tools from experience design such as “customer journey maps” can help to design the experience that is created by deliberately placing design interventions in a particular pattern across an urban context.

### 2.3. Potentials of participatory approaches for urban mental health

In urban development projects for health promotion, participatory approaches have become increasingly important, because aspects of mental health, society, space, and environment share entangled relations (WHO, 2016; UN-Habitat and WHO, 2020). The term *participation* is a widespread and frequently used term derived from the latin word “participo” which describes “the act of taking part in an event or activity.” Following Arnstein (1969), we argue that the more influence someone has on a decision-making process, the greater his or her participation. As outlined earlier, the urban environment plays an important role in influencing health conditions. This is worth considering particularly against the backdrop of heterogeneous user groups in urban settings. This heterogeneity partly stems from changes in the human life-course, as people of all ages – from young children to elderly people – use public spaces. Considering the prevalence of mental health concerns across all ages (Global Burden of Disease Collaborative Network., 2021), this requires taking into account differing but also similar needs (e.g., addressed in universal design, Steinfeld and Maisel, 2012) in the development and design of interventions for urban mental health. In addition, there is a need to consider marginalized groups (e.g., different cultural backgrounds) and people with specific needs (e.g., neurodiverse people), as vulnerable groups are often insufficiently included in planning processes, leading to an underrepresentation of their needs in the resulting environments (Quilling and Köckler, 2018; UN-Habitat and WHO, 2020). Therefore, participatory processes are of special relevance for the development of health-promoting environments. We argue that these issues could be tackled through an approach that offers new models of participation, while focusing on the built environment and involving people of all ages as well as of marginalized groups – in this way, the intertwined aspects of mental health, space, and society could be addressed.

Critical urban theory has been concerned with issues of power and inequality in cities since the 1960s (Brenner, 2009), and while new participatory approaches are constantly developed, the way in which these methods are designed, arranged, and undertaken create barriers to participation (Kuder, 2016). Arnstein (1969) developed a typology of citizen participation in her essay “A ladder of citizen participation.” The concept of a “ladder” or different successive “levels” is an easy-to-use concept that is applicable in different contexts, therefore, it is well known in the fields of urban planning, social work as well as urban health promotion. The concept was adapted over time to different requirements (e.g., *Circles of Decision*, Wright et al., 2008). Aspects of power still play an important role in participatory approaches today, because vulnerable target groups often do not feel empowered enough to take part in participation offers and sometimes lack the necessary means to articulate their needs and concerns in the way that is offered to them (Quilling and Köckler, 2018). Participatory approaches usually remain on the lower middle of Arnsteins ladder, i.e., on the rung of “informing” or “consultation.” Frequently used methods are surveys, round tables, or fishbowl discussions (Kuder, 2016).

To tackle this shortcoming, the approach of *co-creation* offers a process in which participants jointly develop a solution without being the object of research or interview partners, but creators actively shaping their own environments (Jansen, 2018). Co-creation methods are increasingly used in urban planning (Mahmoud et al., 2021). Methods, e.g., from design research, can thus act as a vital link between urban planning and citizens. Using more practical or creative techniques (e.g., joint mapping of the built environment, photo-elicitation, Ortega-Alcázar and Dyck, 2012) than discursive techniques allows contributions from population groups that are otherwise often excluded from such processes (Leino and Puumala, 2021). Nevertheless, power imbalances need to be constantly addressed to avoid their reproduction (Leino and Puumala, 2021). Citizens’ local knowledge is valuable to identify potentials of the built environment of a city, e.g., favorite places for recreation that otherwise are overlooked. Explicitly gathering the individual preferences of different user groups can contribute to a better understanding of their needs (e.g., due to their cultural background). In general, innovative participatory approaches are multifaceted, from StreetArt Festivals (Allianz Vielfältige Demokratie, 2017) to joint construction of buildings in the public space (Leino and Puumala, 2021). To this end, design research, and in particular the approach of design thinking, offers a wide-ranging assortment of methods (Kumar, 2013; Frazer and Kroll, 2022), in which design as a practice of designing acts as a strategic facilitator that seeks collective solutions through inter- and transdisciplinary processes.

Notably, small-scale interventions instead of large-scale projects offer a wide range of approaches that are cost-effective and quick to implement. The concepts of *urban acupuncture* and *tactical urbanism* both pursue these ideas, other frequently used terms are do-it-yourself urbanism or urban prototyping. While in traditional Chinese medicine acupuncture involves tiny pinpricks to reduce pain, the concept of urban acupuncture uses small scale interventions in specific places to increase liveability in neighborhoods (Lerner, 2003; De Solà-Morales i Rubió et al., 2008; Casagrande, 2020). Lydon et al. (2011) promote a similar approach in the concept of tactical urbanism. Tactical urbanism focuses on small-scale interventions to redesign certain urban areas with the help of locals. The basic idea is to test new concepts on a small scale before scaling up and undertaking significant financial and/or political commitments. Co-creative processes can enrich both concepts in their practical implementation.

## 3. Conceptual framework for fostering urban mental health and mental health literacy

Against the backdrop of the aforementioned interdisciplinary theoretical analysis, we propose a conceptual framework for deploying cities as resources for mental health and mental health literacy. The framework builds upon the idea that urban settings act as both risk factors and resources (see Section “2.1. Psychological perspectives on urban mental health”). Deliberate design interventions that consider current best interdisciplinary evidence and suitable design paradigms can address the resources in an urban context (see Section “2.2. Design principles for promoting health and wellbeing”). In this design process, to address truly the needs of as many groups of citizens as possible, special attention needs to be paid to include especially vulnerable users (see Section “2.3. Potentials of participatory approaches for urban mental health”). Thus, a research-driven, interdisciplinary, evidence-based, and co-creative design process is required that considers and embraces multiple facets of the city’s social fabric.

This conceptual framework consists of two levels: The *output* level illustrates the structural model and proposed effect mechanisms for urban design interventions. The *procedural* level refers to the mode of work and the organizational structure that is needed to co-create respective concepts.

### 3.1. Output level

**FIGURE 1:**
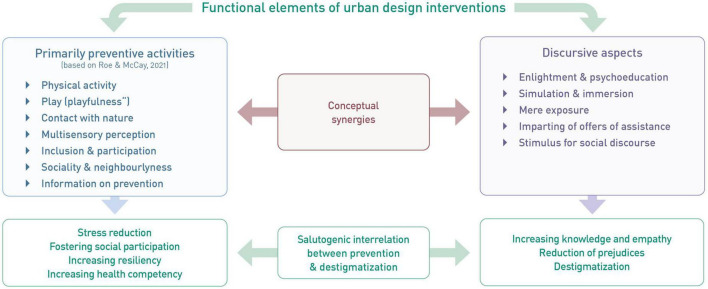
Overview of functional elements and their synergies to foster mental health and mental health literacy in the urban context.

Based on the presented theoretical background from (environmental) psychology, design research, and urban planning, we propose a set of interactive structures that are placed in specific locations in the urban context and directly refer to or even make use of their direct surroundings. These incorporate two main functions (Figure 1): (a) primarily preventive activities, (b) discursive aspects. At this point, we emphasize that examples illustrated in this chapter should only be seen as impulses and serve the purpose of stimulating further thinking and ideating without precluding context-sensitive design. Apart from that, we deliberately remain on an abstract level of analysis without introducing one specific intervention in order to keep the framework applicable to a wide range of design interventions and contexts.

First, building on the *Restorative Cities Framework* (Roe and McCay, 2021), design interventions in the urban context can offer or stimulate activities that proved to be beneficial for mental health and wellbeing. These include for example physical activity (Tamminen et al., 2020), exercises in mindfulness (Davidson et al., 2003), contact with nature (e.g., Alvarsson et al., 2010; Ryan et al., 2014; Salonen et al., 2022), and social interaction (Umberson and Montez, 2010). Additionally, urban design interventions can provide information on prevention of mental health issues such as stress reduction. The actual design of the given intervention and the activity addressed largely depend on the urban context at hand. Especially interventions that guide users’ views or incorporate existing urban greenery by stimulating mindfulness, the urban context itself is the key element of the intervention and should therefore be carefully chosen and integrated. For instance, the growing body of literature on implementing and utilizing nature and the urban context for health benefits illustrates the wide range of design considerations when addressing health by design interventions (Beatley, 2016; Barron et al., 2019; Nieuwenhuijsen et al., 2022). Similar to the ideas of Wolf and Brinkley (2016), a systemic perspective is recommended when applying small scale design interventions to foster mental health. Both in scientific literature as well as realized urban practice, there is a broad range of examples of small-scale interventions as mentioned above. For instance, focusing mainly on physical activity and health in their project PREhealth, Halblaub Miranda et al. (2019) present a number of interventions aligned on a temporary fitness track in the city of Darmstadt. With regards to social interaction and creative stimuli, curated public street art projects combine a number of benefits outlined above. In the last decade, urban gardening initiatives and projects (e.g., Müller I. et al., 2022) illustrated the synergies of participatory approaches that fostered both ecological as well as social improvements for cities. The same applies to pocket parks and public fitness and play installations. Rendering otherwise neglected spaces such as building gaps into micro parks or offering low-threshold opportunities to work out or play mitigates local economic injustices and fosters not only physical health but stimulates social interactions and feelings of belonging and participation. In their Project “Stadtflucht,” Halblaub Miranda and Knöll (2016) make use of augmented reality tools to turn urban spaces into activity and puzzle games. By doing so, both social interaction and the element of playfulness (Graham and Burghardt, 2010) can contribute to mental health and wellbeing.

**FIGURE 2:**
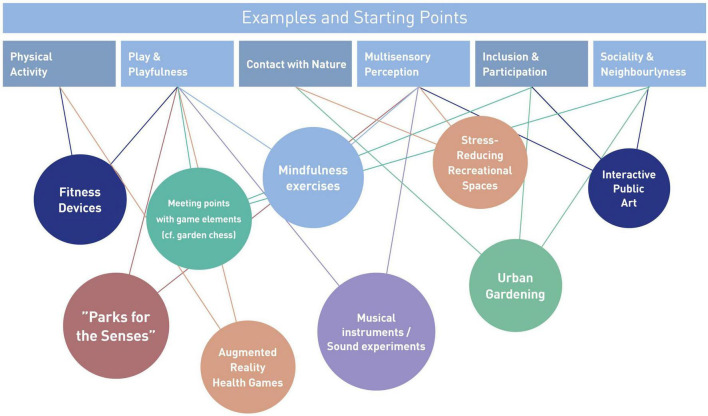
Examples of starting points for small-scale urban design interventions.

Figure 2 illustrates a number of examples or starting points for small scale interventions and their relation to categories outlined in Figure 1. Some of these examples merely refer to activities (e.g., “mindfulness exercise”) without indicating a specific physical representation. These activities could for instance be addressed by visual cueing such as written or visualized instructions. Other examples are typically placed rather in rural or natural settings (e.g., “Parks of the Senses” are usually playground style sets of artifacts that stimulate multisensory interactions in parks or alongside beaches or lakes). However, we argue that these concepts represent enormous potential for the urban contexts when adapted to the specific spatial context. These urban settings pose a number of practical, regulatory and social requirements that need to be addressed when transferring these concepts. Therefore, close collaboration between public and urban designers and authorities is recommended.

Secondly, interventions can address discursive elements such as mental health literacy. Providing information on the emergence, relations, and possible treatment of mental health problems in the public space instead of behind closed doors holds the potential to increase mental health literacy on a low-threshold level. Addressing mental health in such an accessible way and people being confronted with the subject in their everyday lives – both subconsciously (through the mere exposure of the design interventions on their way to work, to the supermarket, or to the playground) as well as consciously through explicit involvement in the interventions (e.g., reading of information) – may achieve normalization and thus lose the connotation of a taboo. In addition, better understanding symptoms of mental health problems might reduce stigmas through information (Corrigan and O’Shaughnessy, 2007). Aspects of simulation and immersion could further increase empathy with those affected and at the same time increase awareness for mental health issues in oneself and one’s surroundings. This element requires special attention to develop content that acknowledges the heterogenous user groups of urban public spaces, including people across all ages, from different cultural backgrounds as well as people facing mental and physical challenges. As with the preventive activities, a number of examples for addressing discursive elements can be found in scientific literature and practice. To facilitate learning processes regarding mental health, gamification approaches such as quizzes can be both useful and low-threshold. Furthermore, presenting small exercises that allow readers to directly experience for instance the interrelation between physical and psychological processes can be additionally persuasive. As an example, simple breathing protocols (e.g., “box breathing,” see Balban et al., 2023) can be both educational and stress reducing. With regards to communication design, apart from graphic and textual illustrations physical models can further help understand general principles and increase curiosity of passersby. Apart from that, information in the public space on existing offerings of therapy, counseling, and support groups can help foster visibility. Furthermore, both activities and information can increase coping skills of people living with mental health problems. With this element, we explicitly go beyond other frameworks (e.g., Roe and McCay, 2021; Bornioli and Subiza-Pérez, 2022) mainly focusing on restoration. Both functions of the proposed interventions pose potential synergistic effects.

To find appropriate locations for such urban design interventions, we advocate a selection of suitable locations based on research and participatory processes. Explorative interviews and walk-along interviews can serve as means to direct researchers’ attention to otherwise overlooked urban scenery. Ideas of changing perspectives on parking lots, old factory premises, or even cemeteries rooted in research on urban green (Kaplan et al., 1998; Harnik, 2012) may be transferred to other types of interventions in the urban context (see Figure 2) and thus foster the selection process. Also, the use of public participation geographic information systems (PPGIS) provides an approach for engaging marginalized groups through integrating and visualizing local knowledge in the form of (interactive) maps. Mapping emotions, behaviors, or activities in certain places can add to contextualize complex spatial information (Sieber, 2006) as a basis for detecting potential for small-scale design interventions, e.g., indicated through informal use. Furthermore, an evidence-based approach can help identify urban resources such as contact to nature (e.g., Ryan et al., 2014; Ryan and Browning, 2020) or views that promote prospect and refuge (Petherick, 2000). Like this, interventions can make use of their direct surroundings.

### 3.2. Process level

**FIGURE 3:**
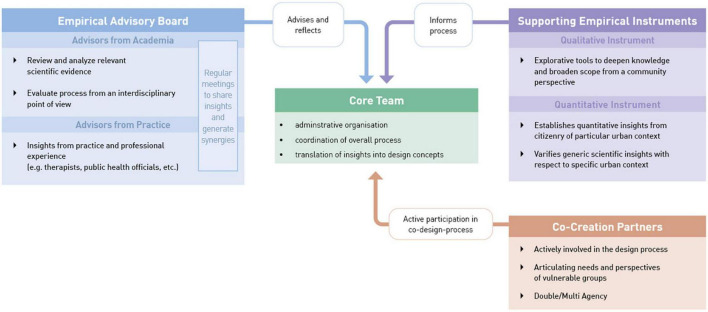
Structural model of the research and design process.

As stated before, in order to co-create effective urban design interventions that aim at fostering mental health for a broad range of users a particularly participatory approach is needed. At the same time, interdisciplinary scientific knowledge needs to be considered to make use of current best evidence. Based on these considerations, we propose a structural model that is built of four elements (Figure 3): At the core of this setup is the administrative and organizational management of the process, which we label as *Core Team*. Without implying any form of hierarchy, the task of this structural element is to coordinate the overall process and translate insights from all other parts into implementable design concepts. From a disciplinary point of view, it is advisable to incorporate experts for public design or other built environment specialists that provide sufficient know-how regarding design processes and the pragmatic needs and requirements of the public space.

In addition, the *Co-Creation Partners* represent a set of relevant stakeholders (e.g., from municipality, NGOs, and representatives from population groups). Public participation broadly distinguishes between three groups that need to be involved in participatory approaches: individuals, citizens’ initiatives, and organized public (such as interest groups, associations). Interest groups and initiatives differ depending on the particular topic (Arbter, 2009). Representatives from different target groups or communities of interest are previously defined as relevant stakeholders through *Stakeholder Mapping* (de Vincente Lopez and Matti, 2016). This group is actively involved in creating new concepts and evaluating them based on their particular role in the project. It is worth mentioning that people can act as “double agents” (Smizz and Walters, 2018) in the sense that they do not only represent one specific group (e.g., with certain vulnerabilities) but relate to other categories as well (Cameron and Grant-Smith, 2005). Participants can therefore, e.g., represent people living with mental health problems and commuters at the same time.

The structural element of an *Empirical Advisory Board* serves to provide guidance to the core team. Interdisciplinary advisors from academia review and analyze the relevant scientific evidence that needs to be considered for designing in the context at hand. Advisors from practice enrich the scientific evidence with experiential insights. The advisory board members meet regularly to evaluate the status of the project based on their synergetic expertise. From a methodological point of view, this advisory board needs to form a mode of work that allows the sharing of knowledge across and even beyond disciplines, which might require the establishment of a specific non-disciplinary lingo. To avoid mere multidisciplinary instead of inter- and transdisciplinary cooperation (Institute of Medicine., 2005), an integration of knowledge is needed, thus achieving truly new insights (Boix Mansilla et al., 2016). The rationale behind this element is the belief that for addressing complex issues such as urban mental health we should not rely on a single discipline but integrate expertise across disciplines (Gruebner et al., 2017) and if possible, across nations.

Finally, *Supporting Empirical Instruments* aim at informing the process by establishing insights from the citizenry of the chosen urban context. In doing so, citizens can be asked on design preferences, usage intentions, and existing knowledge, e.g., to validate initial drafts of urban design interventions. We assess this element to be necessary to translate generic scientific insights to the unique urban context at hand. Here, we distinguish qualitative methods (e.g., focus groups, walk-along interviews) from quantitative methods (e.g., surveys). Insights gained from applying both kinds of methods can complement each other in the design process.

A number of empirical tools and methods can be utilized in line with the quantitative instrument to evaluate the *status quo* of the urban environment at hand: The survey tool “StadtRaumMonitor” (“CitySpaceMonitor,” translated by authors, Bundeszentrale für gesundheitliche Aufklärung, 2023) published by the German Federal Center for Health Education covers 15 constructs clustered into four categories (mobility; public space; supply, work and housing; social interaction). Comparably, the Scottish Government, Architecture and Design Scotland and NHS Health Scotland have developed the Place Standard tool (Our Place Team, 2023) as a simple and participatory evaluation tool for public places. These and other approaches can represent an empirical basis, upon which the participatory process of developing urban design interventions can take place.

## 4. Discussion

This paper aimed at integrating perspectives from environmental and health psychology, design research, and urban planning leading to a conceptual framework for urban design interventions in public space to act as biopsychosocial resources for urban mental health and mental health literacy in a low-threshold, resource-efficient way. On an output level, the framework presents preventive as well as discursive measures, thus including multisensory as well as cognitive engagement. On a process level, it depicts the interplay of different stakeholder groups to be included in the design process, ranging from researchers, to citizenry, as well as municipality. In doing so, we suggest a rich potential of urban environments to act as resources for mental health and to address mental health in the public space (van der Wal et al., 2021). Drawing on interdisciplinary literature and empirical findings, we first illustrated strong associations between mental wellbeing and environments along with the idea to use this relation through deliberate design interventions. In these considerations, we underlined the importance of co-creation in urban design interventions and described the possibilities as well as the pitfalls of participatory approaches. We conclude by discussing considerations for the application of our framework as well as limitations of our work.

### 4.1. Considerations on application of framework

As a pretest of the framework, in November 2022, we presented a simplified version of this framework to an interdisciplinary group of public health experts at the European Public Health Conference (Müller H. et al., 2022). In a workshop format, participants then interactively applied the framework to existing mental health issues in the urban context by referring to resources the city offers. The resulting ideas mainly addressed preventive aspects such as social participation and contact with nature, while discursive aspects were scarce. This may point to the benefit of an inter- and transdisciplinary development of interventions explicitly focusing on the twofold potentials described in the presented framework. While this workshop did not aim at a scientific validation of this framework, *ad hoc* concepts developed by subgroups of the audience showed both innovative and feasible approaches that addressed several considerations presented above. The overall feedback of the interdisciplinary group of experts from fields such as medicine, public health, and social sciences was positive and emphasized the relevance of the topic and innovative potential of this framework.

However, mental health is a sensitive topic that might act as a trigger for certain people. Therefore, it is crucial to include people with lived experiences in the design process of urban design interventions, especially when they entail simulation and immersion. In addition, mental healthcare professionals should be included in creating information material (e.g., psychoeducation) to ensure scientific validity. As the interventions discussed would be placed in urban public spaces, thus offering access to a wide range of people (e.g., in terms of age and educational background), the information provided should be edited in a way that is easily understandable and visually appealing at the same time. Here again, including people of different ages and backgrounds in the design process can increase accessibility to a wide range of users.

To successfully implement such interventions, we argue that citizens should be included in the research process addressing measures to foster mental health in urban environments (Dooris and Heritage, 2013). In doing so, special efforts are required to include hard to reach target groups (e.g., people with low income). Researchers in such processes are required to unlearn their habitual way of doing research and welcome citizens from different backgrounds as experts of their cities.

Further, to successfully implement design interventions for fostering mental health and mental health literacy a close cooperation with the given municipality is needed. As there are various regulations to be considered in urban space, we recommend including municipality stakeholders from the very beginning of a project. Also, design interventions in public space are prone to vandalism, which should be taken into account. However, including citizens in the design process can help reduce vandalism through higher identification with the environment created (Brown et al., 2004). In addition to that, paradigms such as *Design against Crime* (e.g., Armitage and Ekblom, 2019) can be applied. Highlighting that the intended interventions should be cost-efficient and make use of already existing resources in the urban environment may facilitate dialogue with municipality stakeholders.

The proposed design interventions aim to increase prevention but also knowledge and thus agency regarding mental health in the general population. This is in line with other initiatives such as the Mental Health First Aid (MHFA) programme (Kitchener and Jorm, 2008), although in a less formal, institutionalized way. Instead, we propose especially low-threshold measures in public urban spaces to increase awareness for mental health as it gains importance in urban environments (Okkels et al., 2017). An extension of preventive measures with a low threshold is crucial to ensure accessibility by large shares of the urban population. In addition, reaching the largest possible share of the population is a prerequisite to contribute to destigmatization of the topic, as it allows enlightenment not only for a select few. To that aim, these measures need to be decentralized across the city in order to be reachable by people with reduced action range, as well. Further, we suggest to extent mental health literacy to empower people to be attentive to mental health issues, especially in times of crisis such as pandemic, war, and climate change, and to help reduce stigmatization through better information (Corrigan and O’Shaughnessy, 2007). Here, we expect changes in awareness through mere exposure to the topic in day-to-day life. As urban environments are complex systems, interdisciplinary perspectives (e.g., from urban planning, design, and psychology) can help create measures addressing this potential including existing urban features by using evidence-based, salutogenic design.

While this framework addresses resources and implementation protocols for the urban setting, it is likely that this approach is as useful for rural contexts, as well. Still, due to its density of people and offerings, cities pose a particularly rich playing field to pilot this concept.

### 4.2. Limitations and future research

Until now, the proposed framework remains on a conceptual level. Further elaboration for specific contexts and empirical validation is still needed to verify the assumed relations and effects of respective design interventions on citizens’ mental health as well as their mental health literacy. To that aim, single small-scale design interventions could be put up in public spaces as experiments. Accompanying research (e.g., observations, cultural probes, and surveys) including a post-occupancy evaluation (e.g., Preiser and Nasar, 2008) would provide further insights into effectiveness of and reaction to possible interventions. So far, the framework provides an easily applicable approach to fostering mental health and mental health literacy in the urban context by inter- and transdisciplinary cooperation in an innovative way.

Importantly, the proposed urban design interventions are not meant to act as substitutes for psychotherapeutic treatment but could rather have a preventive (and potentially complementary) effect regarding mental disorders and could provide low-threshold information services when needed. The shortage of psychotherapeutic care cannot be solved by such interventions but needs to be tackled on a political level. Yet, contributing to a prevention or mitigation of mental disorders is an important asset addressed here.

## 5. Conclusion

Urban mental health is an increasing challenge of our time. To tackle this challenge, we aim for combining evidence-based, salutogenic, psychosocially supportive design to help increase awareness for mental health–instead of mainly physical health–by implementing low-threshold options in the existing urban context. To this end, inter- and transdisciplinary cooperation deems necessary as urban mental health is a complex topic, which requires the expertise from science as well as lived experience. With this innovative approach, we advocate physically bringing the topic of mental health to the built urban environment to raise awareness, contribute to a destigmatization of the topic and potentially foster mental health and mental health literacy. This promotes the idea that the urban built environment cannot only be restorative, but it also holds the potential to be informative as well as engaging in terms of mental health and mental health literacy for large shares of the population. As public spaces belong to the citizens, they are well used for fostering citizens’ health. This illustrates new directions of research for urban person-environment interactions, public health, and beyond.

## Data availability statement

The original contributions presented in this study are included in the article/supplementary material, further inquiries can be directed to the corresponding author.

## Author contributions

HM: conceptualization, writing–original draft, and writing–review and editing. JR-G: conceptualization, visualization, and writing–original draft. AW: conceptualization and writing–original draft. All authors contributed to the article and approved the submitted version.
